# Incomplete Retinal Pigment Epithelium and Outer Retinal Atrophy Characterized Using Fundus Autofluorescence: An 18-Month Post Hoc Analysis of GATHER2

**DOI:** 10.1167/iovs.67.8.46

**Published:** 2026-07-22

**Authors:** Giulia Corradetti, Aditya Verma, Sophiana Lindenberg, Muneeswar Gupta Nittala, Swetha Velaga, Ayesha Karamat, Ye He, Deniz Oncel, Kenneth Marion, Dina Abulon, SriniVas Sadda

**Affiliations:** 1 Doheny Eye Institute, Pasadena, California, United States; 2 Department of Ophthalmology, David Geffen School of Medicine at UCLA, Los Angeles, California, United States; 3 Department of Ophthalmology and Vision Sciences, University of Louisville, Kentucky, United States; 4 Astellas Pharma Inc., Northbrook, Illinois, United States

**Keywords:** age-related macular degeneration (AMD), geographic atrophy (GA), fundus autofluorescence (FAF), incomplete retinal pigment epithelial and outer retina atrophy (iRORA), complete retinal pigment epithelial and outer retina atrophy (cRORA)

## Abstract

**Purpose:**

To identify fundus autofluorescence (FAF) characteristics corresponding to incomplete retinal pigment epithelial and outer retina atrophy (iRORA), defined using optical coherence tomography (OCT), and progression of iRORA to complete lesions (cRORA).

**Methods:**

In this GATHER2 (NCT04435366) post hoc analysis, pooled eyes from participants assigned to avacincaptad pegol (2 mg) or sham were analyzed for baseline iRORA beyond the borders of geographic atrophy; each iRORA was assessed for progression to cRORA at months 6, 12, and 18 using OCT. For each OCT-defined lesion at baseline and at each follow-up visit, FAF patterns were defined as none (no autofluorescence abnormalities), not classifiable, increased autofluorescence (IAF), questionably decreased (QDAF), or definite decreased (DDAF).

**Results:**

Overall, 153 iRORA from 95 eyes were identified at baseline, and the majority demonstrated none (34.6%; *n* = 53) or QDAF (34.6%; *n* = 53) patterns. A smaller proportion of baseline iRORA exhibited a DDAF pattern (7.2%; *n* = 11). At month 18, the proportion of iRORA manifesting QDAF and DDAF patterns increased to 38.1% and 23.0%, respectively, and the none pattern decreased to 16.8%. The majority of lesions that remained as iRORA at month 18 demonstrated either none (52.8%) or QDAF (49.1%). iRORA that progressed to cRORA by month 18 was more likely to have more advanced FAF abnormalities, with 44.4% having IAF, 30.2% QDAF, and 27.3% DDAF.

**Conclusions:**

Findings from this large study reporting FAF characteristics of iRORA highlight the heterogeneity of these lesions and the association of the multimodal phenotype on progression over time.

A key feature of age-related macular degeneration (AMD) is the presence of extracellular deposits of lipid and protein debris known as drusen, found between the retinal pigment epithelium (RPE) and Bruch's membrane.^1^^–^^4^ Geographic atrophy (GA), characterized by loss of the RPE, the choriocapillaris, and photoreceptors, represents a late stage of AMD and is a leading cause of irreversible central vision loss in adults > 55 years of age.^5^ GA progression can be monitored through a variety of imaging techniques, such as optical coherence tomography (OCT) and fundus autofluorescence (FAF), the current gold standard for GA imaging in clinical trials.^6^^–^^9^ FAF-defined GA includes regions of definite decreased autofluorescence (DDAF) that are > 175 µm in diameter.^10^ FAF also facilitates characterization of the junctional ring surrounding GA, and several FAF junctional patterns have been identified on the basis of the distribution and appearance of the increased autofluorescence signal.^11^^–^^13^ Evidence suggests that the different patterns in FAF imaging in individuals with GA may reflect different stages of the disease and subsequently different risks for disease progression.^11^^,^^12^^,^^14^^,^^15^ Diffuse patterns, particularly the diffuse nontrickling subtype, are associated with faster disease progression, whereas the focal and none patterns progress more slowly.^16^^,^^17^ These patterns can also evolve over time, with one study reporting about 22% of eyes showing a transition in perilesional FAF pattern,^16^ underscoring their value as dynamic biomarkers of GA activity.

The Classification of Atrophy Meeting (CAM) group introduced standardized OCT-based definitions to characterize atrophy. In 2018, the term *complete RPE and outer retina atrophy* (cRORA) was defined, followed in 2020 by *incomplete RPE and outer retina atrophy* (iRORA) to describe earlier atrophic changes considered precursors to cRORA.^18^^,^^19^

iRORA can have a variable phenotype on OCT depending on its severity, appearance, and extension. OCT-defined iRORA lesions correspond to regions of signal hypertransmission into the choroid < 250 µm, with attenuated or disrupted areas of the RPE < 250 µm in diameter (with no evidence of RPE tear), with or without persisting basal laminar deposits, and with signs of overlying photoreceptor degeneration.^18^ By definition, iRORA is any lesion that cannot be classified as cRORA, resulting in a considerably broad spectrum. The iRORA structural characteristics visualized on OCT B-scans might carry a different risk for progression to late-stage AMD or cRORA. The heterogeneity of iRORA lesions can also explain the different rates of progression from iRORA to cRORA that have been reported.^19^ Consequently, a more granular classification of iRORA that takes into consideration the full spectrum of these lesions is necessary, and multimodal imaging may help identify lesions with a higher risk of progression to cRORA or GA.

Positive findings from clinical trials resulted in the first approvals of pegcetacoplan and avacincaptad pegol in 2023 for the treatment of GA secondary to AMD.^20^^–^^23^ These therapeutic advances substantially reduce the rate of GA growth; however, early detection of GA lesions before foveal involvement is key to preserving vision in people living with non-neovascular AMD. Therefore, identification and early detection of iRORA, which is considered the direct precursor of cRORA on OCT, especially the early detection of these iRORA FAF phenotypes with high risk for progression, are pivotal to inform future studies and improve the design of early intervention clinical trials. FAF patterns specifically observable at the iRORA stage—and their predictive value for progression—remain incompletely characterized. The aim of this study was to describe sub-phenotypes of OCT-defined iRORA lesions and determine the association between iRORA-related FAF patterns and progression to cRORA over 18 months.

## Methods

### Study Design

The methodology for the GATHER2 study (NCT04435366) has been described previously.^23^ Briefly, GATHER2 was a randomized, double-masked, sham-controlled phase 3 study evaluating the efficacy and safety of intravitreal injections of avacincaptad pegol (2 mg) versus sham. Participants were eligible if they were ≥ 50 years of age with non-centerpoint GA, with a Snellen best-corrected visual acuity between 20/25 (80 Early Treatment Diabetic Retinopathy Study [ETDRS] letters) and 20/320 (25 ETDRS letters) in the study eye. Participants were ineligible on screening if they did not meet specific ophthalmic inclusion criteria or if they met ophthalmic exclusion criteria, which included GA lesions involving the foveal centerpoint, total GA area size not meeting requirements, and evidence of macular neovascularization in either eye. Avacincaptad pegol has been evaluated in an open-label extension study (NCT05536297) of GATHER2.

The study adhered to the tenets of the Declaration of Helsinki and the International Conference on Harmonisation Good Clinical Practice guidelines. The appropriate ethics committee or institutional review board at each study center approved the protocol. An independent data-monitoring committee reviewed patient safety data during the trial. Written informed consent was obtained from all participants.

### Baseline Assessment of iRORA

At the baseline visit, pooled eyes from the GATHER2 study (participants with GA assigned to either avacincaptad pegol [2 mg] or sham) were assessed for the presence and number of iRORA lesions located at least 500 µm from the GA border. iRORA lesions were identified on the OCT B-scan based on the CAM consensus criteria and marked on the infrared (IR) image using the built-in caliper, thus the distance from the iRORA lesion and the closest border of GA (cRORA previously identified on the OCT B-scan) was measured as a linear distance. Lesion location was marked on the corresponding IR image and registered to the FAF image using the SPECTRALIS built-in image registration tool (Heidelberg Engineering, Heidelberg, Germany), allowing accurate spatial correspondence between OCT-identified lesions and FAF features across visits. Corresponding FAF assessments were also conducted at baseline for each OCT-defined iRORA lesion. The area of the iRORA lesions was not measured, and FAF patterns were qualitatively assessed based solely on the FAF appearance. For each lesion identified on OCT at baseline, the FAF pattern was defined as none (no autofluorescence abnormalities, indicative of early iRORA), increased autofluorescence (IAF; corresponding to a hyperautofluorescent signal that indicates an increased autofluorescence), questionably decreased autofluorescence (QDAF; corresponding to lesions ≤ 175 µm with decreased autofluorescence), or definite decreased autofluorescence (DDAF; corresponding to lesions > 175 µm with decreased autofluorescence suggestive of late iRORA, close to cRORA criteria^24^). DDAF was defined as one or more well-demarcated areas of decreased autofluorescence where the reduction in autofluorescence was at least 90% of the intensity level of the optic nerve or retinal vessels. Lesions exhibiting mixed hyper- and hypo-autofluorescence that could not be confidently assigned to any category were classified as “not classifiable.”

Lesions from both avacincaptad pegol–treated and sham-treated eyes were included; however, grading and analyses were performed under treatment masking. In eyes with multiple iRORA lesions at baseline, lesions were analyzed independently in an exploratory, lesion-level natural history framework. Eyes with baseline iRORA lesions were also examined for the presence of known biomarkers of GA progression such as high-risk drusen (apical height > 45 µm), intraretinal hyperreflective foci, and subretinal drusenoid deposits.^25^^–^^28^

### Evaluation of Progression

Both OCT and FAF data were securely transferred to the Doheny Image Reading and Research Lab for masked analysis. For each iRORA lesion detected on structural OCT, the corresponding findings on autofluorescence imaging and GA peri-junctional background patterns were also assessed. Each iRORA lesion was reassessed at months 6, 12, and 18 using OCT to determine if possible progression to cRORA had occurred. Any iRORA lesions that progressed to cRORA at follow-up were classified as “new cRORA,” and any cRORA that progressed from iRORA at a previous follow-up visit was classified as “persistent cRORA.” Within this post hoc analysis iRORA and cRORA were defined using the CAM group OCT-based definitions to characterize atrophy by the presence of hypertransmission into the choroid ≥ 250 µm, attenuation and/or disruption of the RPE ≥ 250 µm, and associated photoreceptor degeneration, in the absence of an RPE tear. iRORA was defined as lesions demonstrating similar OCT characteristics but not meeting all criteria required for cRORA. Corresponding FAF assessments were conducted for each OCT-defined iRORA lesion at the same time points to determine whether there were any changes to the FAF patterns described above.

### Image Acquisition

In this post hoc analysis of GATHER2, spectral-domain OCT scans were obtained with the Heidelberg SPECTRALIS system or CIRRUS (Carl Zeiss Meditec, Dublin, CA, USA). SPECTRALIS scans were obtained with a 97-line volume scan protocol (20° × 20°, high-resolution mode, automatic real-time [ART] = 9). Scanning with CIRRUS OCT was performed using the 512 × 128 macular cube scan pattern. Blue-light FAF was obtained using the modified three-field imaging protocol (field 1 M, 30° field centered on the temporal aspect of the optic nerve; field 2, 30° field centered on the foveal center; field 3 M, 30° field centered at a location 1–1.5 disc diameters temporal to the center of field 2). FAF images were captured using the SPECTRALIS or Heidelberg Retinal Angiography (HRA) system.^29^

### Grading Strategy

Grading was performed using a predefined protocol based on consensus definitions of iRORA and cRORA and standardized structural OCT criteria. The objective of this study was not to characterize FAF junctional patterns of GA but rather to describe the FAF appearance specifically at locations where iRORA was detected on OCT. All graders involved in this study were experienced and certified Doheny Image Reading and Research Laboratory (DIRRL) graders. Grading was conducted independently by a primary trained grader with senior oversight. In cases of uncertainty or disagreement, images were reviewed jointly, and final adjudication was performed by the director of the DIRRL to ensure consistency and adherence to protocol. Prior to study initiation, graders participated in protocol calibration sessions using representative cases to harmonize interpretation of lesion boundaries and biomarker definitions.

### Statistics

Descriptive statistics (counts and percentages) were used to summarize baseline characteristics, as well as progression at months 6, 12, and 18. Given the exploratory nature of the analysis and the relatively small sample size, no formal inferential or hypothesis-driven statistical testing was performed; analyses were descriptive and intended to generate hypotheses.

## Results

### Baseline Analyses

**Figure 1.**
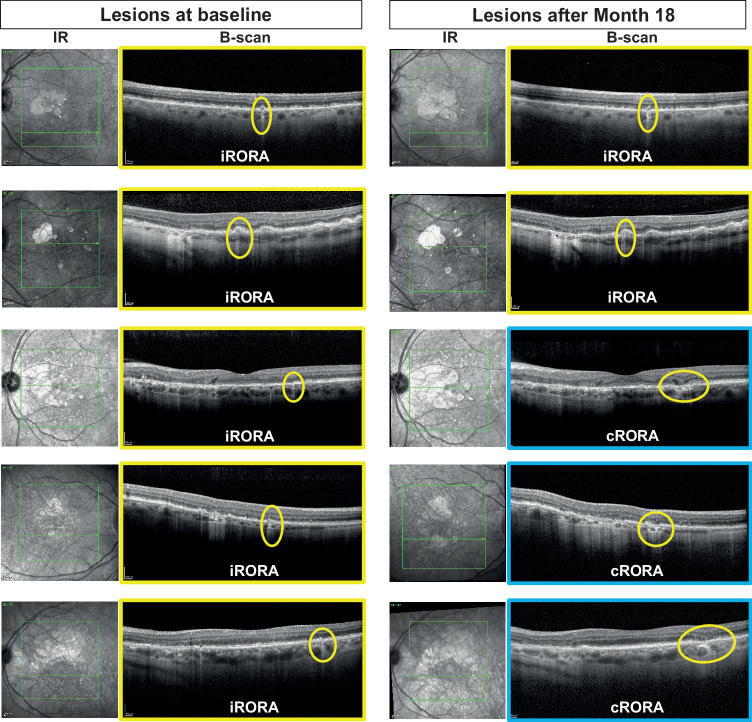
Representative IR and OCT B-scans of lesions identified at baseline and corresponding scan data collected at month 18. Shown are representative baseline IR and OCT B-scans (*left panels*) and corresponding scan data collected at month 18 (*right panels*) to assess the progression of baseline iRORA lesions at each follow-up. iRORA lesions are characterized by a heterogeneous spectrum, from early changes with minimal hypertransmission defects to more advanced lesions with large hypertransmission defects. Consequently, these differences may reflect on the FAF pattern and might affect the rate of progression. The *yellow circles* are used to localize iRORA and cRORA lesions within the OCT B-scan.

**Figure 2.**
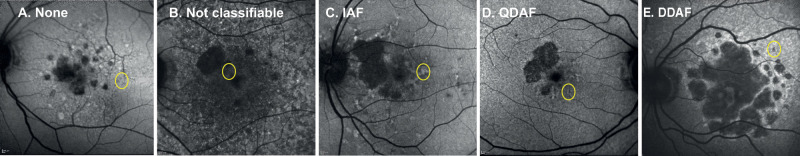
FAF patterns of OCT-defined iRORA lesions at baseline. The *circle* indicates an area showing the FAF pattern described in the respective panel. (**A**) None, corresponding to the absence of a distinct increased or decreased autofluorescence pattern. (**B**) Not classifiable, corresponding to the presence of a not distinct pattern of increased or decreased autofluorescence. (**C**) IAF, corresponding to a hyperautofluorescent signal that indicates an increased autofluorescence. (**D**) QDAF, corresponding to the presence of a decreased signal that does not meet the criteria for DDAF for reflectivity or size. (**E**) DDAF, corresponding to the presence of a definite hypoautofluorescent signal.

OCT data from 448 GATHER2 participants were examined. A total of 153 iRORA lesions from 95 eyes were identified at baseline and presented a wide variety of phenotypes (Table 1). Approximately 85% of eyes (81 of 95) had one to two iRORA lesions per eye on OCT at baseline. Figure 1 (left panels) shows representative images of iRORA lesions assessed at baseline using OCT. Among the 153 OCT-defined iRORA lesions, most were associated with none or QDAF patterns (*n* = 53 [34.6%] each), nine lesions (5.9%) showed an IAF pattern, and 11 lesions (7.2%) showed a DDAF pattern (Table 1). Representative FAF images of OCT-defined iRORA lesions assessed at baseline are shown in Figure 2.

**Table 1.** Cross-Sectional Analysis of iRORA Lesions at Baseline

Characteristic	Value
iRORA lesions (on OCT), *n*	153
Eyes with iRORA lesions (on OCT), *n*	95
iRORA lesions per eye (on OCT), *n* (%)	
1	58 (61.1)
2	23 (24.2)
3	9 (9.5)
4	4 (4.2)
>5	1 (1.1)
FAF characteristic of baseline iRORA (identified on OCT), *n* (%)	
None	53 (34.6)
Not classifiable	27 (17.6)
IAF	9 (5.9)
QDAF	53 (34.6)
DDAF	11 (7.2)
Distance from DDAF to GA border (µm), mean ± SD	2387.4 ± 514.3

High-risk drusen (apical height > 45 µm) were identified in 105 iRORA lesions (68.6%) using OCT (Table 2). Ninety-seven of these lesions (92.4%) were present in eyes with more than five high-risk drusen (with a mean apical height of > 100 µm). Over 70% of the baseline iRORA lesions were identified in eyes with intraretinal hyperreflective foci (134 of 153 lesions; 87.6%) and in eyes with subretinal drusenoid deposits (110 of 153 lesions; 71.9%).

**Table 2.** OCT Assessment of High-Risk Structural Features in Eyes With Baseline iRORA Lesions

Feature	Value
Total iRORA lesions at baseline, *n*	153
Total high-risk drusen (>45 µm in height), *n* (%)	105 (68.6)
iRORA in eyes with the indicated number of high-risk drusen, *n* (%)	
1	4 (3.8)
2	3 (2.9)
3	0
4	1 (1.0)
>5	97 (92.4)
Maximum apical height of high-risk drusen (µm), mean ± SD	102.0 ± 31.6
Intraretinal hyperreflective foci, *n* (%)	134 (87.6)
Subretinal drusenoid deposits, *n* (%)	110 (71.9)
Drusen with hyporeflective core, *n* (%)	71 (46.4)

### iRORA Progression

**Figure 3.**
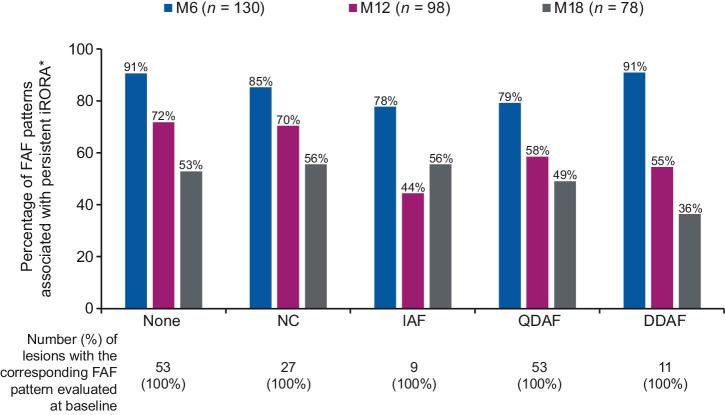
FAF patterns associated with iRORA lesions that remained as iRORA at follow-up visits (persistent iRORA). *The denominator is the total number of lesions with the corresponding FAF pattern evaluated at baseline. M, month.

The trends in iRORA progression were evaluated over time using OCT and FAF. Among the 153 locations where baseline iRORA were detected, we were able to assess 134, 126, and 113 of these locations at months 6, 12, and 18, respectively, as follow-up locations where the OCT quality was not optimal were not assessed. (Supplementary Table S1). The number of iRORA detected at the same baseline location on OCT decreased over time, with 130 of 134 lesions (97.0%) identified at month 6, 98 of 126 lesions (77.8%) at month 12, and 78 of 113 lesions (69.0%) at month 18. Regarding the progression of iRORA to cRORA, the greatest rate of cRORA lesion onset was recorded at month 12, with 23 of 126 new lesions (18.3%) identified compared with four of 134 new lesions (3.0%) at month 6 and 11 of 113 new lesions (9.7%) at month 18. By month 18, the cumulative conversion rate was 24.8% (*n* = 38) (Supplementary Table S2). The number of persistent cRORA (progressed at previous follow-up visit) increased over time, with none of 134 lesions (0%) identified at month 6, five of 126 lesions (4.0%) at month 12, and 24 of 113 lesions (21.2%) at month 18. By month 18, the number of iRORA decreased by 49% and the number of new onset cRORA nearly increased by three-fold. Representative OCT images collected at month 18 are shown in Figure 1 (right panels). Evaluation of FAF phenotypes at baseline iRORA locations showed that the proportion of lesions exhibiting a none FAF pattern declined from 27.6% (37/134) at month 6 to 16.8% (19/113) at month 18, and QDAF increased from 34.3% (46/134) to 38.1% (43/113) over the same period. Notably, DDAF showed a marked increase from 11.2% (15/134) at month 6 to 23.0% at month 18. In parallel, the proportion of not classifiable lesions decreased over time. The IAF pattern remained infrequent, accounting for less than 4% of FAF patterns at all assessed time points. The majority of lesions that remained as iRORA (persistent iRORA) at month 18 demonstrated either a none pattern (28 of 53 lesions; 52.8%) or a QDAF pattern (26 of 53 lesions; 49.1%) (Fig. 3; Supplementary Table S3).

**Figure 4.**
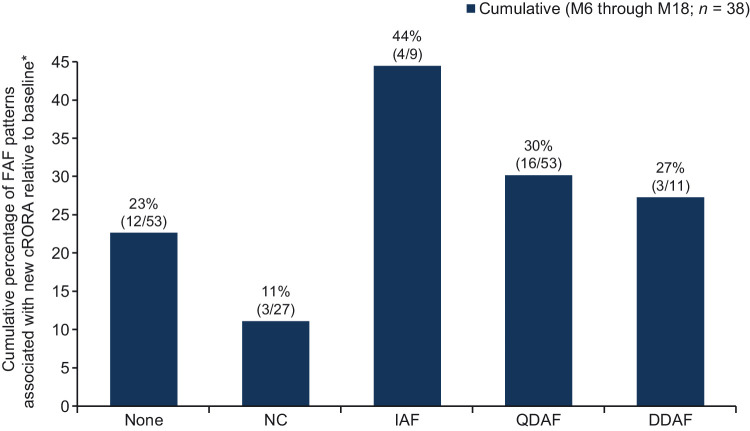
Cumulative proportions of FAF patterns associated with iRORA progression to cRORA at follow-up visits (new cRORA). *The denominator is the total number of lesions with the corresponding FAF pattern evaluated at baseline.

iRORA lesions that progressed to cRORA by month 18, with a progression rate of 24.8% (38 of 153 lesions), were more likely to have more advanced FAF abnormalities, with four of nine lesions (44.4%) having IAF, 16 of 53 lesions (30.2%) having QDAF, and three of 11 lesions (27.3%) having DDAF (Fig. 4; Supplementary Table S2).

## Discussion

This post hoc study analyzed OCT and FAF imaging data from the GATHER2 trial to characterize iRORA lesion phenotypes and their progression in patients with GA secondary to AMD. At baseline, most iRORA lesions were associated with high-risk drusen, intraretinal hyperreflective foci, or subretinal drusenoid deposits, known structural features associated with fast GA progression.^25^^–^^28^ Moreover, iRORA lesions identified by OCT at baseline corresponded to a wide spectrum of FAF patterns. This finding highlights the opportunity to potentially differentiate those patterns into early or late iRORA phenotypes, in line with previous reports underlining the importance of multimodal imaging for therapeutic interventions focusing on retinopathies.^30^^,^^31^ By further characterizing the phenotype of baseline iRORA using FAF, the findings reported here may help researchers to better understand the natural history of these lesions and to achieve a more granular classification of these lesions as a precursor to atrophy. Additionally, this may help clinicians with early diagnosis while also helping to optimize the design of early intervention clinical trials. More importantly, these findings may assist eyecare professionals with identifying FAF characteristics of OCT-defined iRORA, a level of granularity not yet supported in the literature. The early identification of FAF features corresponding to OCT-defined iRORA supports early decision-making processes to potentially maximize slowing of GA progression and vision loss.

Over the 18-month follow-up, the number of iRORA lesions on OCT decreased over time, whereas the number of persistent cRORA lesions increased. Lesions transitioned from iRORA to cRORA, with corresponding FAF patterns shifting from none to QDAF and DDAF. Persistent iRORA lesions most often were none or QDAF patterns; over time the none pattern frequency decreased, whereas QDAF increased, indicating progression from early to mid-stage iRORA. Lesions progressing to cRORA frequently exhibited QDAF and DDAF patterns, suggesting more advanced disease (early cRORA). Persistent cRORA lesions (lesions who progressed at the previous visit), increasingly showed DDAF, suggesting lesion enlargement (persistent cRORA). Advanced FAF patterns may be suggestive of higher cRORA progression; however, these correlations are speculative and should be considered hypotheses, particularly in discussing persistent cRORA lesions, which were not measured in the analyses. Moreover, it should be noted that progression rates from iRORA to cRORA have varied in different populations, but the focus of this analysis was to correlate OCT and FAF structural findings corresponding to these lesions.

As precursors to GA, understanding the natural history of iRORA lesions remains crucial for predicting disease progression and developing effective therapeutic interventions. However, the knowledge of the natural history of intermediate AMD and iRORA is limited. A better understanding of the natural history of iRORA lesions may lead to a more granular classification of intermediate AMD, which is needed to optimize the design of early intervention clinical trials. Additionally, it is important for patient stratification based on risk factors, which would support clinicians in assessing the most suitable course of care.

Key limitations of this study are the exploratory nature of the analysis, the small sample size, and the short follow-up time. In addition, multiple lesions per eye were analyzed using lesion-level descriptive statistics without adjustment for intra-eye correlation or consideration of treatment group, as the study is a post hoc analysis and was not designed or powered to evaluate clustering effects or treatment-related influences on iRORA lesion evolution. Formal inferential statistical testing was not performed; therefore, observed differences in FAF phenotype frequencies over time should not be interpreted as statistically significant or clinically meaningful trends. Despite these limitations, this is one of the first studies, to our knowledge, to characterize FAF imaging patterns corresponding with OCT-defined iRORA and cRORA lesions in participants with GA from the randomized, double-blind phase 3 GATHER2 study. This is of particular interest, as a complete representation of the changes occurring in AMD could only be detected with OCT thus far.^24^

These observations align with prior reports demonstrating that perilesional FAF patterns are not static and that certain hyper- and hypo-autofluorescent phenotypes are associated with faster progression.^16^^,^^17^ The consistency with previous FAF studies supports the potential value of integrating multimodal imaging including both OCT and FAF to facilitate a more granular classification of iRORA and may help better determine the timing of treatment options for these lesions. Early identification of atrophic changes may help eyecare professionals make informed treatment decisions to slow disease progression, help preserve photoreceptors, and help preserve vision.

Collectively, these findings suggest that integrating OCT and FAF imaging can improve characterization of the natural history and risk profiles of atrophic AMD lesions. Sub-phenotyping iRORA based on FAF patterns may offer clinically meaningful insights to support earlier identification of lesions at higher risk of progression, inform patient monitoring strategies, and refine the design of future clinical trials. Importantly, the ability to recognize risk factors and precursors to atrophy remains critical to enabling timely diagnosis, closer follow-up, and informed discussions around emerging treatments aimed at slowing GA growth before foveal involvement and irreversible vision loss occur. Although the exploratory design, limited sample size, and relatively short follow-up warrant caution in generalizing these results, the observations underscore the potential value of early detection and multimodal imaging in improving GA management and guiding future research.

## Supplementary Material

Supplement 1

## References

[1] Fleckenstein M, Mitchell P, Freund KB, et al. The progression of geographic atrophy secondary to age-related macular degeneration. *Ophthalmology*. 2018; 125(3): 369–390. 29110945 10.1016/j.ophtha.2017.08.038

[2] Holz FG, Strauss EC, Schmitz-Valckenberg S, van Lookeren Campagne M. Geographic atrophy: clinical features and potential therapeutic approaches. *Ophthalmology*. 2014; 121(5): 1079–1091. 24433969 10.1016/j.ophtha.2013.11.023

[3] Boyer DS, Schmidt-Erfurth U, van Lookeren Campagne M, Henry EC, Brittain C. The pathophysiology of geographic atrophy secondary to age-related macular degeneration and the complement pathway as a therapeutic target. *Retina*. 2017; 37(5): 819–835. 27902638 10.1097/IAE.0000000000001392 PMC5424580

[4] Ferris FL 3rd, Wilkinson CP, Bird A, et al. Clinical classification of age-related macular degeneration. *Ophthalmology*. 2013; 120(4): 844–851. 23332590 10.1016/j.ophtha.2012.10.036 PMC11551519

[5] Guymer RH, Campbell TG. Age-related macular degeneration. *Lancet*. 2023; 401(10386): 1459–1472. 36996856 10.1016/S0140-6736(22)02609-5

[6] Cheng AMS, Chalam KV, Brar VS, et al. Recent advances in imaging macular atrophy for late-stage age-related macular degeneration. *Diagnostics (Basel)*. 2023; 13(24): 3635. 38132220 10.3390/diagnostics13243635 PMC10742961

[7] Heesterbeek TJ, Lorés-Motta L, Hoyng CB, Lechanteur YTE, den Hollander AI. Risk factors for progression of age-related macular degeneration. *Ophthalmic Physiol Opt*. 2020; 40(2): 140–170. 32100327 10.1111/opo.12675 PMC7155063

[8] Holz FG, Sadda SR, Staurenghi G, et al. Imaging protocols in clinical studies in advanced age-related macular degeneration: recommendations from Classification of Atrophy consensus meetings. *Ophthalmology*. 2017; 124(4): 464–478. 28109563 10.1016/j.ophtha.2016.12.002

[9] Pfau M, Künzel SH, Pfau K, Schmitz-Valckenberg S, Fleckenstein M, Holz FG. Multimodal imaging and deep learning in geographic atrophy secondary to age-related macular degeneration. *Acta Ophthalmol*. 2023; 101(8): 881–890. 37933610 10.1111/aos.15796 PMC11044135

[10] Velaga SB, Nittala MG, Hariri A, Sadda SR. Correlation between fundus autofluorescence and en face OCT measurements of geographic atrophy. *Ophthalmol Retina*. 2022; 6(8): 676–683. 35338026 10.1016/j.oret.2022.03.017

[11] Bindewald A, Schmitz-Valckenberg S, Jorzik JJ, et al. Classification of abnormal fundus autofluorescence patterns in the junctional zone of geographic atrophy in patients with age related macular degeneration. *Br J Ophthalmol*. 2005; 89(7): 874–878. 15965170 10.1136/bjo.2004.057794 PMC1772741

[12] Holz FG, Bellman C, Staudt S, Schütt F, Völcker HE. Fundus autofluorescence and development of geographic atrophy in age-related macular degeneration. *Invest Ophthalmol Vis Sci*. 2001; 42(5): 1051–1056. 11274085

[13] Holz FG, Bindewald-Wittich A, Fleckenstein M, et al. Progression of geographic atrophy and impact of fundus autofluorescence patterns in age-related macular degeneration. *Am J Ophthalmol*. 2007; 143(3): 463–472. 17239336 10.1016/j.ajo.2006.11.041

[14] Schmitz-Valckenberg S, Bültmann S, Dreyhaupt J, Bindewald A, Holz FG, Rohrschneider K. Fundus autofluorescence and fundus perimetry in the junctional zone of geographic atrophy in patients with age-related macular degeneration. *Invest Ophthalmol Vis Sci*. 2004; 45(12): 4470–4476. 15557456 10.1167/iovs.03-1311

[15] Wolf-Schnurrbusch UE, Enzmann V, Brinkmann CK, Wolf S. Morphologic changes in patients with geographic atrophy assessed with a novel spectral OCT–SLO combination. *Invest Ophthalmol Vis Sci*. 2008; 49(7): 3095–3099. 18378583 10.1167/iovs.07-1460

[16] Antropoli A, Vacirca F, Introini U, Bandello F, Battaglia Parodi M, Cicinelli MV. Perilesional fundus autofluorescence patterns are not static: longitudinal transitions in geographic atrophy and association with disease progression. *Ophthalmol Sci*. 2026; 6(2): 100995. 41439216 10.1016/j.xops.2025.100995 PMC12719152

[17] Biarnés M, Arias L, Alonso J, et al. Increased fundus autofluorescence and progression of geographic atrophy secondary to age-related macular degeneration: the GAIN study. *Am J Ophthalmol*. 2015; 160(2): 345–353.e5. 25982972 10.1016/j.ajo.2015.05.009

[18] Guymer RH, Rosenfeld PJ, Curcio CA, et al. Incomplete retinal pigment epithelial and outer retinal atrophy in age-related macular degeneration: classification of Atrophy Meeting Report 4. *Ophthalmology*. 2020; 127(3): 394–409. 31708275 10.1016/j.ophtha.2019.09.035 PMC7218279

[19] Corradetti G, Corvi F, Nittala MG, et al. Natural history of incomplete retinal pigment epithelial and outer retinal atrophy in age-related macular degeneration. *Can J Ophthalmol*. 2021; 56(5): 325–334. 33539821 10.1016/j.jcjo.2021.01.005

[20] Heier JS, Lad EM, Holz FG, et al. Pegcetacoplan for the treatment of geographic atrophy secondary to age-related macular degeneration (OAKS and DERBY): two multicentre, randomised, double-masked, sham-controlled, phase 3 trials. *Lancet*. 2023; 402(10411): 1434–1448. 37865470 10.1016/S0140-6736(23)01520-9

[21] Apellis. Syfovre prescribing information. Available at: https://pi.apellis.com/files/PI_SYFOVRE.pdf. Accessed October 7, 2025.

[22] Astellas. Izervay prescribing information. Available at: https://www.astellas.com/content/dam/astellas-com/global/en/documents/izervay_pi.pdf. Accessed October 7, 2025.

[23] Khanani AM, Patel SS, Staurenghi G, et al. Efficacy and safety of avacincaptad pegol in patients with geographic atrophy (GATHER2): 12-month results from a randomised, double-masked, phase 3 trial. *Lancet*. 2023; 402(10411): 1449–1458. 37696275 10.1016/S0140-6736(23)01583-0

[24] Sadda SR, Guymer R, Holz FG, et al. Consensus definition for atrophy associated with age-related macular degeneration on OCT: classification of Atrophy Report 3. *Ophthalmology*. 2018; 125(4): 537–548. 29103793 10.1016/j.ophtha.2017.09.028 PMC11366072

[25] Monés J, Garcia M, Biarnés M, Lakkaraju A, Ferraro L. Drusen ooze: a novel hypothesis in geographic atrophy. *Ophthalmol Retina*. 2017; 1(6): 461–473. 31047436 10.1016/j.oret.2017.02.006

[26] Flores R, Carneiro Â, Tenreiro S, Seabra MC. Retinal progression biomarkers of early and intermediate age-related macular degeneration. *Life (Basel)*. 2021; 12(1): 36. 35054429 10.3390/life12010036 PMC8779095

[27] Au A, Santina A, Abraham N, et al. Relationship between drusen height and OCT biomarkers of atrophy in non-neovascular AMD. *Invest Ophthalmol Vis Sci*. 2022; 63(11): 24. 10.1167/iovs.63.11.24 PMC9624265 36306145

[28] Lad EM, Finger RP, Guymer R. Biomarkers for the progression of intermediate age-related macular degeneration. *Ophthalmol Ther*. 2023; 12(6): 2917–2941. 37773477 10.1007/s40123-023-00807-9 PMC10640447

[29] Corradetti G, Karamat A, Srinivas S, et al. Progression to complete retinal pigment epithelium and outer retinal atrophy (cRORA): post hoc analysis of the GATHER1 trial. *Graefes Arch Clin Exp Ophthalmol*. 2024; 263(3): 669–677. 39538001 10.1007/s00417-024-06676-7 PMC11953152

[30] Strauss RW, Muñoz B, Ho A, et al. Progression of Stargardt disease as determined by fundus autofluorescence in the retrospective Progression of Stargardt Disease study (ProgStar Report No. 9). *JAMA Ophthalmol*. 2017; 135(11): 1232–1241. 29049437 10.1001/jamaophthalmol.2017.4152 PMC5710470

[31] Al-Khuzaei S, Shah M, Foster CR, et al. The role of multimodal imaging and vision function testing in ABCA4-related retinopathies and their relevance to future therapeutic interventions. *Ther Adv Ophthalmol*. 2021; 13: 25158414211056384. 34988368 10.1177/25158414211056384 PMC8721514

