# Supplementary material for: Incomplete Retinal Pigment Epithelium and Outer Retinal Atrophy Characterized Using Fundus Autofluorescence: An 18-Month Post Hoc Analysis of GATHER2
Source: Invest Ophthalmol Vis Sci. 2026 Jul 22;67(8):46. doi: 10.1167/iovs.67.8.46 (PMC13401120; doi:10.1167/iovs.67.8.46)
Supplement: Supplement 1 [file iovs-67-8-46_s001.pdf]

## SUPPLEMENTARY INFORMATION

**Table S1.** Assessment of iRORA Progression Using OCT

| | **Month 6, *n* (%)** | **Month 12, *n* (%)** | **Month 18, *n* (%)** |
|---|---|---|---|
| Number of baseline iRORA locations excluded | 19 (12.4) | 27 (17.6) | 40 (26.1) |
| Number of baseline iRORA locations analyzed | 134 (87.6) | 126 (82.4) | 113 (73.9) |
| Persistent iRORA at the same baseline location | 130 (85.0) | 98 (64.1) | 78 (51.0) |
| iRORA progressed to cRORA (new cRORA) | 4 (2.6) | 23 (15.0) | 11 (7.2) |
| Persistent cRORA (progressed at previous follow-up visit) | - | 5 (3.3) | 24 (15.7) |
| Total number of baseline iRORA locations | 153 (100) | 153 (100) | 153 (100) |

cRORA, complete retinal pigment epithelium and outer retinal atrophy; iRORA, incomplete retinal pigment epithelium and outer retinal atrophy; OCT, optical coherence tomography.

**Table S2.** Proportions of FAF Patterns Associated With iRORA Progression to cRORA at Follow-Up Visits (New cRORA) Relative to Baseline

| | | **Proportion relative to baseline** | | | |
|---|---|---|---|---|---|
| | **N at baseline** | **Month 6, *n* (%)** | **Month 12, *n* (%)** | **Month 18, *n* (%)** | **Cumulative*, *n* (%)** |
| All patterns | 153 | 4 (2.6) | 23 (15.0) | 11 (7.2) | 39 (25.5) |
| None | 53 | 1 (1.9) | 6 (11.3) | 5 (9.4) | 12 (22.6) |
| Not classifiable | 27 | 0 (0) | 2 (7.4) | 1 (3.7) | 3 (11.1) |
| IAF | 9 | 2 (22.2) | 1 (11.1) | 1 (11.1) | 4 (44.4) |
| QDAF | 53 | 1 (1.9) | 12 (22.6) | 3 (5.7) | 17 (32.1) |
| DDAF | 11 | 0 (0) | 2 (18.2) | 1 (9.1) | 3 (27.3) |

*Month 6 through month 18. cRORA, complete retinal pigment epithelium and outer retinal atrophy; DDAF, definite decreased autofluorescence; FAF, fundus autofluorescence; IAF, increased autofluorescence; iRORA, incomplete retinal pigment epithelium and outer retinal atrophy; QDAF, questionably decreased autofluorescence.

**Table S3.** Assessment of iRORA Progression Using FAF

| | **Month 6** | **Month 12** | **Month 18** |
|---|---|---|---|
| Total baseline iRORA locations evaluated, *n* | 134 | 126 | 113 |
| None, *n* (%) | 37 (27.6) | 28 (22.2) | 19 (16.8) |
| Not classifiable, *n* (%) | 21 (15.7) | 13 (10.3) | 4 (3.5) |
| IAF, *n* (%) | 5 (3.7) | 2 (1.6) | 4 (3.5) |
| QDAF, *n* (%) | 46 (34.3) | 55 (43.7) | 43 (38.1) |
| DDAF, *n* (%) | 15 (11.2) | 18 (14.3) | 26 (23.0) |
| Excluded: CG/NE/NA, *n* (%) | 10 (7.5) | 10 (7.9) | 17 (15.0) |

CG, cannot grade; DDAF, definite decreased autofluorescence; FAF, fundus autofluorescence; IAF, increased autofluorescence; iRORA, incomplete retinal pigment epithelium and outer retinal atrophy; NA, not available/missing data; NE, not evaluable due to poor image quality; QDAF, questionably decreased autofluorescence.
